# Unconventional use of injectable long-acting cabotegravir and rilpivirine against HIV-1 in PWH in clinical need: 52 weeks real-world data

**DOI:** 10.1186/s12879-025-10499-0

**Published:** 2025-01-22

**Authors:** Valentina Iannone, Roberto Rossotti, Nicholas Brian Bana, Gabriele Cavazza, Federico D’Amico, Francesca Lombardi, Pierluigi Francesco Salvo, Gianmaria Baldin, Simona Di Giambenedetto, Dario Bernacchia, Gabriele Pagani, Alberto Borghetti, Stefano Rusconi

**Affiliations:** 1 Department of Healtcare Surveillance and Bioetichs, Section of Infectious Diseases, Catholic University of Sacred Heart, Rome, Italy; 2 Department of Infectious Diseases, ASST Grande Ospedale Metropolitano Niguarda, Milan, Italy; 3 Department of Medical and Surgical Science, Fondazione Policlinico Universitario A. Gemelli IRCCS, UOC Infectious Diseases, Rome, Italy; 4 Clinical and Biomedical Science Department (DIBIC), Infectious Diseases Unit, University of Milan, Legnano General Hospital, ASST Ovest Milanese, Italy, Legnano, 20025 MI Italy; 5 Department of Clinical and Experimental Medicine, University of Pisa, Pisa, Italy

**Keywords:** Long acting antiretrovirals, Population in need, Comorbidities, Adherence issues, Virological suppression

## Abstract

**Background:**

Long-acting Cabotegravir and Rilpivirine (LA CAB + RPV) shows potential advantages in heavily comorbid and even in viremic people with HIV (PWH). We assessed LA CAB + RPV durability in a cohort of PWH with a high comorbidity burden and adherence issues.

**Methods:**

Retrospective observational study in two Italian outpatient settings enrolling PWH who switched to LA CAB + RPV from February 2021 to January 2024 in presence of exclusion criteria enlisted in registrational trials or with other worrisome clinical risks. Kaplan-Meier (KM) was used to assess the probability of CAB/RPV discontinuation. Cox regression analysis was used to evaluate potential predictors of discontinuation.

**Results:**

We enrolled 74 PWH, with a median of 7 injections (IQR 5–9), a median age of 53 years (IQR 45–61), median time of exposure to antiretrovirals of 11 years (IQR 8–18) and median time from HIV diagnosis of 11.8 years (IQR 6.6–18.2). Eleven (14.9%) discontinued LA CAB + RPV mainly for injection-site pain. Of 53 PWH who were undetectable before switch, 37 maintained viral suppression at week 52. We registered only one virological failure at week 12. Twenty-one started injections with unsuppressed viral loads (median 66 cps/ml, IQR 40–215) and 10 (47.6%) achieved viral suppression. Overall probability of discontinuation was 14.9% at week 52. Younger age was protective against discontinuation (HR 0.93, 95%CI 0.88–0.99, *p* = 0.048).

**Conclusions:**

Our results support the potential advantages in using LA CAB + RPV in PWH with adherence issues and comorbidities.

## Background

There have been great expectations on the feasibility and efficacy of long-acting compounds against HIV, now being cabotegravir (CAB) plus rilpivirine (RPV). There will be other options soon, but by now these two intramuscular drugs are the only available options. There is a great interest from people with HIV (PWH) on long-acting treatments as it was demonstrated by previous analyses [1–3]. In this new “injectable era” clinicians are also facing the challenge of a radical renewing, implementation, and reorganization of outpatient clinics, to optimize resources and improve medical and nursing assistance to reach even the most fragile individuals, notwithstanding that this is a difficult goal to achieve on a large scale. Many individuals, probably mainly due to polypharmacy and constrained socio-economic conditions, are still not fully adherent to oral regimens, figuring out a possible new target population for long-agents CAB + RPV [4]. As shown in the compassionate use of LA CAB + RPV [5], there are several potential advantages from long-acting administration for PWH who need an approach different from oral therapies. A list of these benefits include: achieve viral suppression in PWH with persistent detectable low level viremia due to low adherence without resistance associated mutations (RAMs); decrease pill fatigue; overcome swallowing troubles [5]; reduce drug-drug interactions; reduce stigma; allow administration at home for individuals with physical or psychiatric issues who have difficulties reaching the clinic [6]; good safety profile in pregnancy [7]; potential use in teenagers. We have also to be cautious with the potential exit strategies in the case of inefficacy, intolerance or virologic failure of long-acting therapies [8]. Existing literature provides few data about use of LA CAB + RPV in individuals who might benefit from an injectable approach despite complex clinical conditions that could deter clinicians from employing this treatment. Herein we present our data from a cohort of PWH who received LA CAB + RPV and with a high rate of comorbidities and adherence issues, and who had some exceptions at the level of prescribing criteria as reported in the Summary of Product Characteristics (SmPC).

## Methods

This observational retrospective analysis enrolled PWH who switched to LA CAB + RPV from February 2021 to January 2024 in two Italian outpatient settings (ASST Grande Ospedale Metropolitano Niguarda, Milan; Fondazione Policlinico Universitario A. Gemelli IRCCS, Rome, Italy) to overcome medical or social issues that would make other oral options less suitable. The main outcome was to evaluate probability of LA CAB + RPV continuation, rate of virologic failure, and variables associated with LA CAB + RPV discontinuation in this fragile population. To be included, individuals should present at least 2 follow-up injections, some clinical conditions that were enlisted as exclusion criteria in registrational trials (confirmed detectable HIV RNA; major psychiatric diseases; liver cirrhosis; active oncologic disease; solid organ transplant recipient; hemodialysis for end-stage kidney disease; neurologic or oncologic conditions leading to swallowing difficulties; previous failure to first-generation nonnucleoside reverse transcriptase inhibitors). We also included subjects poorly represented in clinical trials or with clinical conditions in need of real-world data such as transgender women (with or without gender affirming hormone therapy but without gluteal implants), elderly PWH older than 65 years. PWH with detectable HIV RNA have been included if repeated viremia values above 50 copies/mL were attributable to low adherence at treating physician’s discretion and not for underlying RAMs affecting cabotegravir and/or rilpivirine fold-change. Enrolled individuals might present one or more inclusion criteria. Although some of these conditions are considered as contraindicated in the drugs SmPC, each case was evaluated by the treating physicians who decided to start LA CAB + RPV for medical or social issues despite such contraindications. Demographic, clinical, virologic and immunologic data were retrieved from hospital electronic records at baseline (BL), and at weeks (W) 4, 12, 28, 52 (4 W, 12 W, 28 W, 52 W). If available, genotypic resistance test and quantitative HIV DNA before the switch were collected. In case of virologic failure (defined as two determinations of HIV RNA > 50 cp/ml or a single determination of HIV RNA > 200 cp/ml) emerging RAMs were registered. Descriptive analysis was conducted to characterize the subjects included in the study. Median values and interquartile ranges (IQR) were used to describe continuous variables, while counts and percentages were employed for qualitative variables. Kaplan-Meier (KM) curves were used to estimate the Probability of CAB + RPV continuation. Univariable Cox regressions were used to analyze the strength of association between LA CAB + RPV continuation and other variables, calculating hazard ratios and their 95% confidence intervals (CI). The sample calculation was based on the primary efficacy endpoint at week 52, the proportion of patients with virologic suppression (HIV RNA < 50 copies/mL). Given the lack of data in the literature on the effectiveness of long-acting therapy in the specific population considered for the study, and assuming a 50% probability of virological suppression at week 52, we estimated that a sample size of at least 68 patients would be required to test our hypothesis, with a 0.10 margin of error and a 90% confidence interval (calculation performed using Epitools online software, available at: https://epitools.ausvet.com.au/oneproportion?OneProportion%5Bproportion%5D=0.5%26OneProportion%5Bprecision%5D=0.10%26OneProportion%5Bconf%5D=0.9%26OneProportion%5Bpopsize%5D=). Two-tailed p-values were calculated and a value < 0.05 was considered statistically significant. Data management and statistical analysis were performed in SPSS version 22.0 (IBM, Chicago, IL, USA). The study was conducted according to the Helsinki declaration. All data used in the study were previously anonymized, according to the requirements of the Italian Data Protection Code (leg. Decree 196/2003) and by the general authorizations issued by the Data Protection Authority. As anonymized data generated from routine clinical practice were retrospectively managed in aggregated form, approval by the Ethics Committee was deemed unnecessary according to the Italian law (art. 6 and art. 9, leg. decree 211/2003).

## Results

**Fig. 1:**
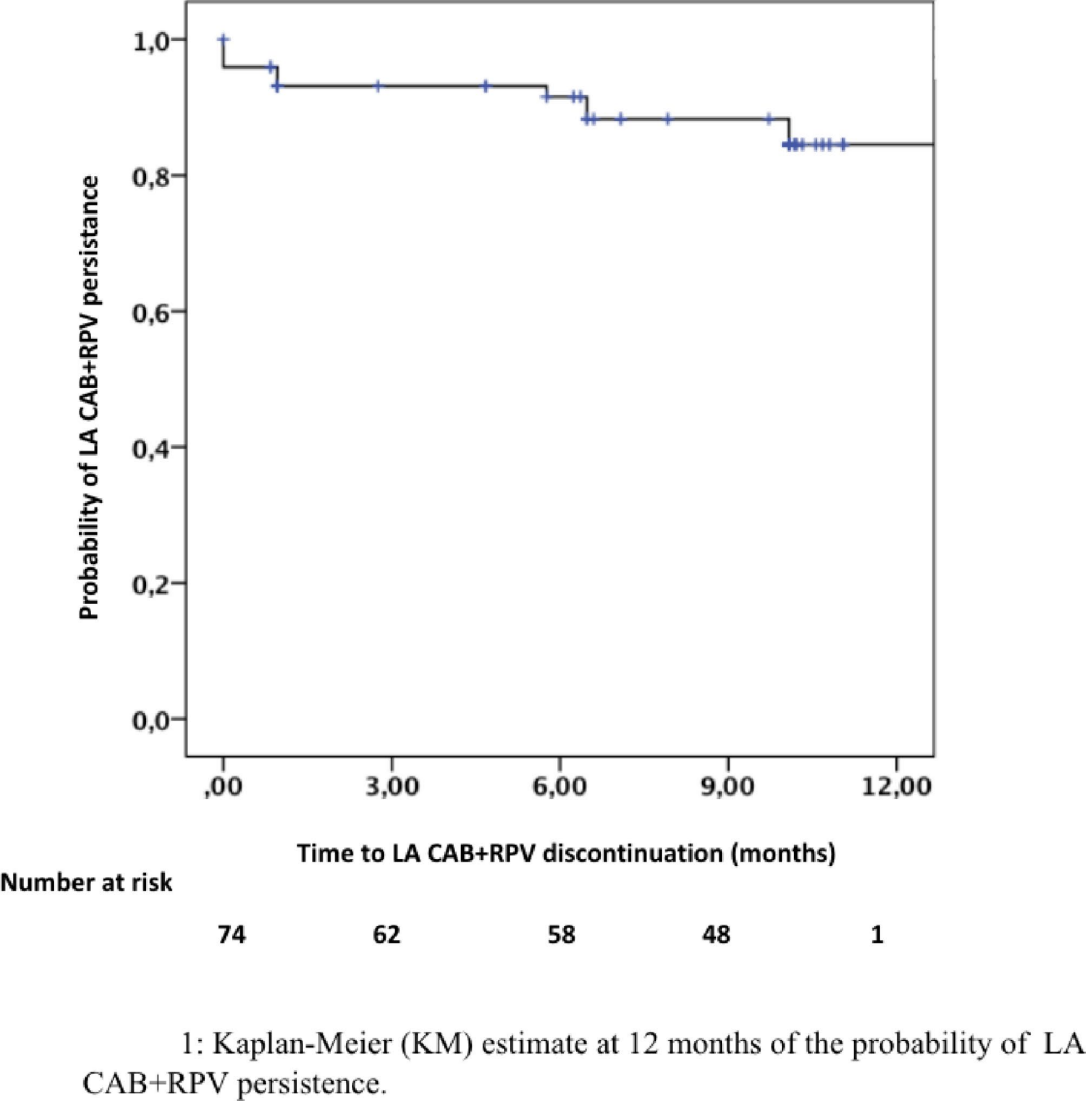
Kaplan-Meier (KM) estimate at 12 month of the probability of LA CAB+RPV continuation

We enrolled 74 PWH, with at least 2 follow-up injections and a median of 7 injections (IQR 5–9). Study population was mainly composed by males (59, 79,7%) with a median age of 54 years (IQR 45–61). The median time of exposure to antiretroviral therapy in this population was of 11 years (IQR 8–18) and the median time from HIV diagnosis was 11,8 years (IQR 6.6–18.2). Thirty-six participants switched from a dual-therapy regimen INI-based (48,6%), while 27 PWH (36,5%) were on a 3-drug regimen INI-based before switching to LA CAB + RPV. Only 7 participants underwent the oral lead in phase (9.5%). Full population characteristics are summarized in Table 1. Ten PWH received injections bimonthly at home by the home care assistance service through a qualified and dedicated nursing service. At BL 26 PWH (35.1%) reported poor adherence to oral daily ART (inability to maintain everyday pill-taking), while 14 participants switched to LA CAB + RPV with a Body Mass Index > 30 Kg/m^2^. Seven individuals reported a diagnosed psychiatric disorder at BL (9,5%), 6 PWH had swallowing disorders with inability to take oral antiretroviral therapies, while one individual was on replacement dialysis treatment (81,4%) at that time of switch. In addition, 39 PWH (52.7%) had ≥2 diagnosed comorbidities at the time of enrolment. Furthermore, NNRTI resistance-associated mutations (RAMs) were detected in twelve participants at BL. Fifty-three (72%) participants had virologic suppression before switch to LA CAB + RPV, while 21 PWH (28.4%) started injections with unsuppressed viral loads (median TCD4 cell count of 594 cells/mm3, IQR, 339–1035: median VL 66 cps/ml, IQR, 40–215). Of 53 PWH who had virologic suppression before switch to LA CAB + RPV and with a median of 7 injections (IQR 5–9), 37 reached 52 weeks of follow up, maintaining viral suppression during the observation time. The remaining 15 participants who did not complete the 52-week follow-up period demonstrated sustained virological suppression at week 28, while one experienced virological failure. Of 21 PWH (28.4%) who started injections with unsuppressed viral loads, with a median of 7 injections (IQR 2-8.5), 11 of them reached 52 W of follow-up and 10 achieved viral suppression. We registered only one virological failure at 12 W (4th injection): HIV viral load was 1170 cp/mL, genotypic resistance testing was performed and showed: N115H and H51Y mutations in the integrase gene and non-polymorphic E138K mutation and LA CAB-RPV was discontinued toward TAF/FTC/DRV/c. The aforementioned participant was 47 years old, male, HIV subtype A1, with TCD4 + lymphocyte nadir of 201 cells/mmc and HIV-RNA Zenith of 32,569 cp/ml; he was on a 3-drug regimen before switching to LA CAB + RPV with DOR/3TC/TDF with virologic suppression (HIV RNA < 30 cp/ml at BL), in the absence of mutation at the previous genotypic resistance test. Notably the BMI was 33.81 Kg/m^2^ at BL (Obesity Class I). Regarding discontinuation of LA CAB + RPV, 11 PWH (15%), discontinued the injectable regimen (after a median of 3 injections) mainly for pain in injection-site (45.5%). Table 2 presents the baseline characteristics of the subset of subjects who discontinued LA CAB + RPV. Three individuals (4%) opted voluntarily to move to another center, while one discontinued for virological failure (1,4%). A total of two cases (2.7%) were attributed to a personal afterthought, a motivation that was not elucidated during the interview with the referring physician. By KM at 52 W after switching LA CAB-RPV, the overall probability of of CAB/RPV continuation was 85.1% (Fig. 1). Younger age was associated with discontinuation (per 1 year increase, HR 0.93 95% CI, 0.88–0.99, *p* = 0.048) by Cox regression (Table 3). No safety issues were recorded during the observation study period.

**Table 1:** Full population characteristic at baseline

Variables	*N* = 74
Age, years, median, (IQR)	54 (44.7–61)
Gender n (%) *Cis-gender man* *Cis-gender woman* *Transgender woman*	59 (79.7) 12 (16.2) 3 (4.1)
Ethnicity, n (%) *Caucasian* *African* *Latino-American*	65 (87.8) 3 (4.1) 5 (6.8)
Risk Factor n (%) *MSM* *Heterosexual* *PWID*	35 (47.3) 28 (37.8) 9 (12.2)
Zenith HIV-RNA as log10 copies/mL, median (IQR)	5.22 (4.48–5.72)
Nadir CD4, cells/mm3, median (IQR)	234.5 (62.5-484.2)
Time since HIV diagnosis, years, median (IQR)	11.8 (6.6–18.2)
Time on ART, years, median (IQR)	11 (8–18)
Antiretroviral regimen before BL, n (%) - 2NRTI + INSTI - 2NRTI + NNRTI - 2NRTI + PI - 2DR (3TC/DTG, RPV/DTG) - Other 2DR - Other ARV regimen	27 (36.5) 2 (2.7) 3 (4.1) 36 (48.6) 3 (4.1) 3 (4.1)
CDC Stage C, n (%)	27 (36.5)
**HIV VL ≤ 30 copies/ml at BL**,** n (%)**	53 (71.6)
**HIV VL ≥ 30 copies/ml at BL**,** n (%)**	21 (28.4)
TCD4 + cells/mm3, median (IQR) at BL	651 (431–1014)
BMI, Kg/m2, median (IQR)	25.4 (22.6–30.2)
Oral lead in, n (%)	7 (9.5)
PWH with NNRTI resistance-associated mutations (RAMs) at BL, n (%)	12 (16%)
Comorbidities, n (%) Cardiovascular disease Endocrinological disorders Hepatology disease Psychiatric disease Neurological disease Pulmonary disease Oncological disease	23 (31.1) 27 (36.5) 27 (36.5) 18 (24.3) 12 (16.2) 2 (2.7) 5 (6.8)
PWH with ≤2 comorbidities, n (%) PWH with ≥2 comorbidities, n(%)	35 (47.3) 39 (52.7)

**Table 2:** Baseline characteristics of participant who discontinued LA CAB + RPV

Variables	*N* = 11
Age, years, median, (IQR)	47 (41–53)
Gender n (%)	
*Cis-gender man*	9 (81.8)
*Cis-gender woman*	1 (9.1)
*Transgender woman*	1 (9.1)
Ethnicity, n (%)	
*Caucasian*	7 (63.6)
*African*	2 (18.2)
*Latino-American*	2 (18.2)
Risk Factor n (%)	
*MSM*	4 (36.4)
*Heterosexual*	7 (63.6)
*PWID*	0 (0)
Zenith HIV-RNA as log10 copies/mL, median (IQR)	4.96 (3.62–5.48)
Nadir CD4, cells/mm3, median (IQR)	360 (201–520)
Time since HIV diagnosis, years, median (IQR)	8 (4.2–14.5)
Time on ART, years, median (IQR)	8 (6–15)
Antiretroviral regimen before BL, n (%)	
- 2NRTI + INSTI	3 (27.3)
- 2NRTI + NNRTI	1 (9.1)
- 2NRTI + PI	0 (0)
- 2DR (3TC/DTG, RPV/DTG)	6 (54.5)
- Other 2DR	1 (9.1)
- Other ARV regimen	0 (0)
CDC Stage C, n (%)	11 (100)
**HIV VL ≤ 30 copies/ml at BL**,** n (%)**	8 (72.7)
**HIV VL ≥ 30 copies/ml at BL**,** n (%)**	3 (27.3)
TCD4 + cells/mm3, median (IQR) at BL	734 (509–1020)
BMI, Kg/m2, median (IQR)	27.2 (23.7–33.6)
Oral lead in, n (%)	1 (9.1)
PWH with NNRTI resistance-associated mutations (RAMs) at BL, n (%)	3 (27.3)
Comorbidities, n (%)	
Cardiovascular disease	3 (27.3)
Endocrinological disorders	5 (45.5)
Hepatology disease	3 (27.3)
Psychiatric disease	1 (9.1)
Neurological disease	0 (0)
Pulmonary disease	1 (9.1)
Oncological disease	0 (0)
PWH with ≤2 comorbidities PWH with ≥2 comorbidities	7 (63.6) 4 (36.4)

**Table 3:** Cox univariate analysis

VARIABLES	HR	CI	*P* value
Age (per 1 year increase)	0.93	0.88–0.99	0.0048
Sex *-Cis-gender woman* *- Cis-gender man* *- Transgender woman*	0 1.85 5.90	0.23–14.8 0.36–96.7	0.563 0.213
Ethnicity *-Caucasian* *- Non Caucsian*	0 1.25	0.40-18.32	0.90
Risk Factor n (%) *MSM* *Heterosexual* *PWID*	0 1.13 0.002	0.32-4.00 0.001–1.03	0.849 0.982
Zenith HIV-RNA (per 1 log10 copies/mL increased)	0.74	0.40–1.38	0.342
Nadir CD4, (per 100 CD4 cell count increase)	1.26	0.98–1.63	0.070
Time since HIV diagnosis (per 1 year increase)	0.93	0.85–1.02	0.112
Time on ART, (per 1 year increase)	0.94	0.86–1.04	0.224
Antiretroviral regimen before BL - Triple therapy - Dual therapy	0 1.62	0.41–6.30	0.484
CDC Stage C	0.024	0.001–4.86	0.168
HIV VL ≤30 copies/ml at BL	0		
HIV VL ≥30 copies/ml at BL	1.27	0.33–4.92	0.731
TCD4 + cells/mm3, median (IQR) at BL	1.01	0.87–1.18	0.835
BMI, (per unit increase)	1.03	0.91–1.16	0.653
Oral lead in	0.04	0.001-140	0.555
PWH with NNRTI resistance-associated mutations (RAMs) at BL	2.74	0.70–10.7	0.146
Comorbidities Cardiovascular disease Endocrinological disorders Hepatology disease Psychiatric disease Neurological disease Pulmonary disease Oncological disease	0.96 1.04 0.76 0.31 0.36 0.05 0.05	0.26-4.00 0.29–3.69 0.19–2.94 0.04–2.44 0.001-38 0.001-inf 0.00-inf	0.962 0.953 0.693 0.266 0.350 0.774 0.571
PWH with ≤2 comorbidities, n (%) PWH with ≥2 comorbidities, n(%)	0 0.34	0.88–1.31	0.118

## Discussion

The data corroborate the potential advantages of employing LA CAB-RPV in PWH with adherence issues and comorbidities. In accordance with the most recent evidence from real-world settings, this unconventional use of injectable CAB + RPV could represent an alternative approach to this strategy, offering a means of overcoming the necessity for strict daily adherence to pill-taking, thereby substantially reducing barriers to adherence. Our goal has been bridging the remaining gaps and reaching PWH most in need. Also, we kept in mind to find the best place for LA therapy. ​​ In the first demonstration project in San Francisco, administration of injectable CAB + RPV in non-suppressed PWH, coupled with extensive social supports, led to a viral suppression rate of 96.4% (55/57) [6], encouraging clinicians to consider the possible use of LA CAB + RPV in this setting. Despite all these listed benefits, deviating from the ideal target population proposed by clinical trials and from the suggested selection criteria, a word of caution must be considered [8]. A significant concern about to the prescription of CAB-RPV to individuals who do not meet the established eligibility criteria is the potential for virologic failure and the emergence of drug resistance in two crucial classes of (ART), INSTIs and NNRTIs [9]. Although trials showed virological failure rate around 1%, the emergence of resistance to both agents has prompted concerns about the viability of subsequent “exit strategies” [8]. In individuals with a long history of HIV infection, a complex medical background and several concomitant medications that could potentially lead to critic drug-drug interactions (DDIs), it is crucial to undertake a comprehensive and proactive assessment of their therapy prior to initiating LA CAB + RPV. This should include a detailed review of the patient’s previous response to a PI-based regimen, particularly if there has been documented intolerance or allergy [10]. In our study population, only 6/74 individuals (8%) switched to LA CAB + RPV from a PI-based regimen, with no reported intolerance before the switch. Given the high prevalence of comorbidities in this setting, further evaluation of potential DDIs is warranted in cases of breakthrough viremia and the necessity of switching to boosted darunavir. It is evident that the extent of adherence to standardized injections must also be considered, as this may result in sub-optimal drug concentrations of CAB + RPV, which in turn may lead to viral breakthrough and acquired drug resistance in viremic PWH. Our study provides information about this more complex population that has never been enrolled in clinical trials and furthermore, this paper presents a prospective management model for home care assistance, which could be implemented in a range of hospital care settings. The potential for administering LA CAB + RPV via a scheduled home visit by a trained nursing staff could prove an effective strategy for addressing poor adherence to daily ART and treatment fatigue, reaching fragile individuals as well as for enhancing patient well-being over time. This highlights the necessity of implementing comprehensive health programs and extensive social support to reach a greater number of comorbid PWH who need assistance, thereby improving outcomes for these individuals and increasing virological suppression rates in this complex population. Our study suffers from some limitations that should be acknowledged, including the generalizability of the data presented from our bicentric cohort. Additional clinical research is necessary to corroborate these findings with a larger sample size and a longer follow-up. This will assist in defining the actual target population for this promising long-acting injectable strategy.

## Conclusions

Data from our bicentric cohort depict clinical experiences with unconventional use of injectable LA CAB + RPV and might provide a suitable venue for this strategy to reach PWH in need.

## Data Availability

The datasets used and/or analyzed during the current study are available from the corresponding author on reasonable request. Valentina Iannone, Roberto Rossotti, and Stefano Rusconi should be contacted if someone wants to request the data from this study.

## Abbreviations

PWH: People with HIV
LA CAB + RPV: Long-acting cabotegravir and rilpivirine
KM: Kaplan-meier
PrEP: Pre-exposure prophylaxis
CAB: Cabotegravir
RPV: Rilpivirine
BMI: Body mass index
RAMs: Resistance associated mutations
